# Internal Stark effect of single-molecule fluorescence

**DOI:** 10.1038/s41467-022-28241-8

**Published:** 2022-02-03

**Authors:** Kirill Vasilev, Benjamin Doppagne, Tomáš Neuman, Anna Rosławska, Hervé Bulou, Alex Boeglin, Fabrice Scheurer, Guillaume Schull

**Affiliations:** Université de Strasbourg, CNRS, IPCMS, UMR 7504, F-67000 Strasbourg, France

**Keywords:** Atomic and molecular interactions with photons, Electronic properties and materials, Excited states

## Abstract

The optical properties of chromophores can be efficiently tuned by electrostatic fields generated in their close environment, a phenomenon that plays a central role for the optimization of complex functions *within* living organisms where it is known as internal Stark effect (ISE). Here, we realised an ISE experiment at the lowest possible scale, by monitoring the Stark shift generated by charges confined within a single chromophore on its emission energy. To this end, a scanning tunneling microscope (STM) functioning at cryogenic temperatures is used to sequentially remove the two central protons of a free-base phthalocyanine chromophore deposited on a NaCl-covered Ag(111) surface. STM-induced fluorescence measurements reveal spectral shifts that are associated to the electrostatic field generated by the internal charges remaining in the chromophores upon deprotonation.

## Abstract (web summary)

The internal Stark effect, a shift of the spectral lines of a chromophore induced by electrostatic fields in its close environment, plays an important role in nature. Here the authors observe a Stark shift in the fluorescence spectrum of a phthalocyanine molecule upon charge modifications within the molecule itself, achieved by sequential removal of the central protons with a STM tip.

## Introduction

In many chemical and biological systems, the electric fields generated by embedded electrostatic charges regulate the absorption or emission energies of chromophores.^1–3^. This phenomenon, known as internal Stark effect (ISE),^3–5^ contrasts with Stark shifts induced by external electric fields^6,7^. It is at play in natural light-harvesting complexes where the sensitive optical properties of chlorophyll^8,9^ and carotenoid^2,10^ are adjusted by local charges in surrounding proteins and neighboring compounds to enable energy funneling. Similarly, electrostatic interactions between retinal chromophores and neighboring charged groups are responsible for wavelength regulation of vision^1^. In these examples, ISE occurs in complex landscapes composed of a large number of interacting organic systems. Scaling down these effects to single-molecules would be a step towards understanding the intimate interaction between biological pigments and their electrostatic environment, but has been so far limited to the spectroscopy of molecules in frozen matrices^11–14^ and scanning-tunneling microscopy (STM) experiments^15–18^ where *external* electric fields were used to shift the molecular states. The effect of an electric field generated by an elementary charge located within a chromophore on its optical properties remains up to now a Gedankenexperiment. Here, we use free-base phthalocyanine (H_2_Pc) molecules, deposited on a NaCl-covered silver sample, as a model system to study the ISE induced by one or two charges localised at the center of the chromophore on its fluorescence properties. To do so, we successively remove the two central protons of a H_2_Pc molecule. STM images, topographic time traces, and differential conductance spectra are used to identify the nature of the deprotonated species and their electronic structure. STM-induced luminescence (STML) spectra recorded on the singly and doubly deprotonated compounds reveal a fluorescence emission blue-shifted compared to the original H_2_Pc chromophore. Based on a comparison with time-dependent density functional theory (TD-DFT) simulations, we show that these shifts can be traced back to the radial electric field generated by charges confined to the *σ*-orbitals of the deprotonated chromophores whereas their *π*-orbitals remain unchanged. As a consequence, the neutral and deprotonated compounds are iso-electronic, in contrast with scanning probe experiments in which charging a molecule alters its *π*-orbitals^19–24^.

The deprotonation procedure also affects the vibronic emission of the molecule, inducing measurable frequency shifts for several modes, an effect that is discussed in terms of a vibrational Stark effect^25^ and mass changes similar to isotopic shifts. Overall, our experiment constitutes an ultimate ISE experiment where the electric field is generated directly inside the probed chromophore, a model landmark for more complex ISE-induced color-tuning phenomena occurring in biological systems, and a novel strategy to develop tunable optoelectronic devices relying on single molecules as active components.

## Results

### Deprotonation of H_2_Pc molecules on NaCl/Ag(111)

**Fig. 1:**
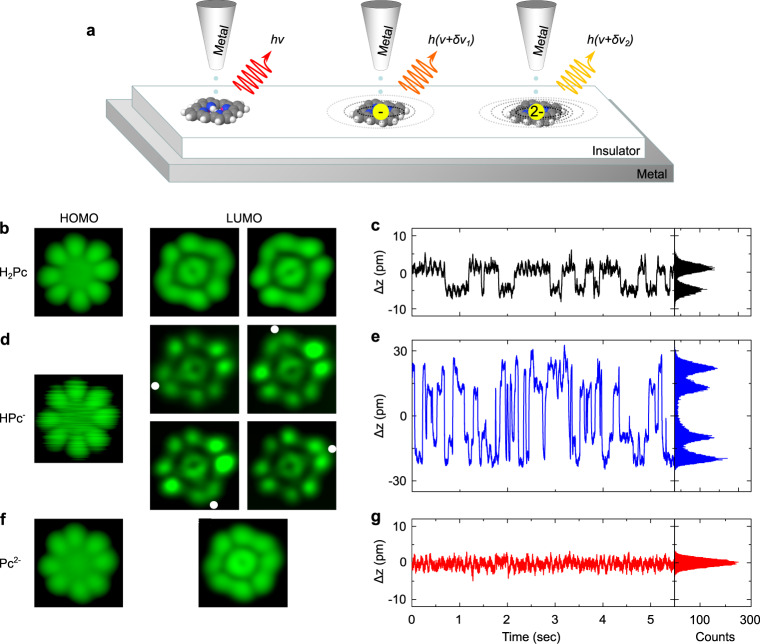
Imaging protonated and deprotonated phthalocyanine molecules and monitoring tautomerization processes. **a** Sketch of the experiment where the fluorescence energy (*hν*) of a phthalocyanine molecule is progressively Stark-shifted (*δhν*_i_) by charges located at the center of the chromophore. **b**, **d**, **f** STM images (2.7 × 2.7 nm^2^) of a phthalocyanine molecule on 3ML-NaCl/Ag(111). The images of the HOMO were all recorded at *I*  = 10 pA, with voltage *V* = −2.5 V for H_2_Pc, *V* = −1.5 V for HPc^−^ and *V* = −2 V for Pc^2−^. The LUMO were recorded at *I* = 10 pA, *V* = 0.5 V for (**b**) H_2_Pc, *I* = 2.3 pA, *V* = 1.43 V for (**d**) HPc^−^ and *I* = 10 pA, *V* = 1.65 V for (**f**) Pc^2−^. The LUMO images of H_2_Pc and HPc^−^ reveal two and four different patterns, respectively, in successive images. The dots in the LUMO images of HPc^−^ indicate the side where the remaining hydrogen atom is located. **c**, **e**, **g** Relative tip-sample distance (Δz) time traces and their histograms recorded at a bias voltage of −2.5 V on (**c**) H_2_Pc, (**e**) HPc^−^, and (**g**) Pc^2− ^, and at constant current (I_set−point_ = 10 pA for H_2_Pc  and 5 pA for HPc^−^ and Pc^2−^).

Figure 1a shows a sketch of the STM-induced luminescence (STML) experiment used to probe the Stark effect generated by central charges on the fluorescence of a phthalocyanine chromophore deposited on a NaCl-covered Ag(111) surface (see “Methods” for details). To realise this scheme, we worked with H_2_Pc molecules whose typical STM images recorded at *V* = −2.5 V and *V* = 0.5 V are displayed in Fig. 1b. While the left image reveals the characteristic four-fold symmetry pattern of the highest occupied molecular orbital (HOMO), the right one reflects the twofold symmetry pattern of the lowest unoccupied molecular orbital (LUMO) (see Supplementary Figs. 1–3 and Supplementary Table 1). This latter can adopt two configurations rotated by 90^∘^ from each other in successive images^26,27^, a behavior that has been formerly assigned to tautomerization, i.e., the permutation of the central hydrogen atoms between two equivalent sites of the molecule. This phenomenon can be tracked in the variation of the tip-sample distance (Δz) versus time (Fig. 1c) recorded at constant current where it appears as two-level fluctuations^24,27–31^. As a next step of the experiment, we located the tip on top of the center of the H_2_Pc molecule and applied a positive voltage ramp at a constant current of 10 pA while simultaneously recording the relative tip-sample distance “z”. In most cases, this procedure reveals a sudden “z” decrease for *V* ≈ 3.2 V (see Supplementary Note 1 for details), hinting towards a change of the molecular structure. STM images recorded at *V* = −1.5 V (HOMO) after such an event are similar to the one measured before, but are fuzzier (Fig. 1d). Δz time-traces now reveal four-level fluctuations with a high switching frequency (Fig. 1e). This explains the fuzzy appearance of the HOMO image and hints towards the inner motion of a single proton in a HPc molecule^24,29,31^. This is confirmed by STM images acquired at *V* = 1.43 V [LUMO, (Fig. 1d)] where each tautomer can be stabilized and identified. The voltage is then ramped a second time until another sudden distance drop occurs. The typical four-fold symmetry STM images (Fig. 1f) observed for both HOMO and LUMO of the resulting compound^32^, together with non-fluctuating Δz time traces (Fig. 1g) now suggest a doubly dehydrogenated (or deprotonated) phthalocyanine molecule (Pc).

### Electronic structure of the deprotonated H_2_Pc molecules

**Fig. 2:**
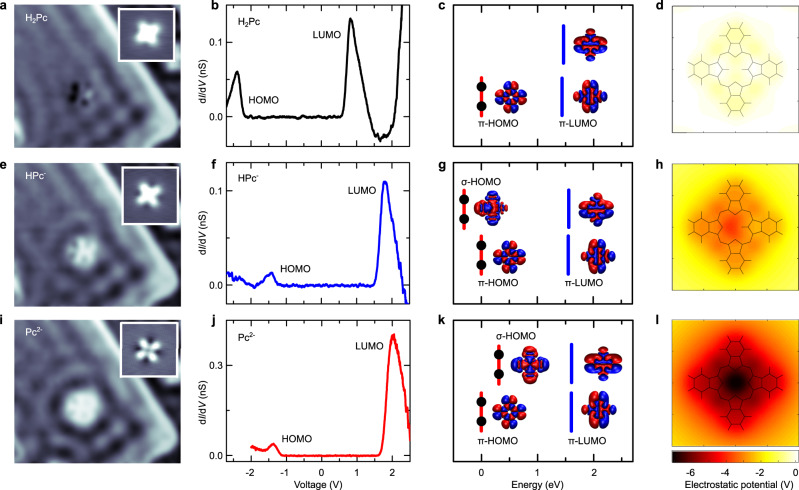
Identifying charges confined in deprotonated phthalocyanines. **a**, **e**, **i** Constant current d*I*/d*V* maps (*I* = 30 pA, *V* = 400 mV, 12.5 × 10.8 nm^2^, modulation voltage *δV*_mod_ = 50 mV) recorded on (**a**) H_2_Pc, (**e**) HPc^−^, and ﻿(**i**) Pc^2−^. Images (**a**), (**e**), and (**i**) were acquired on a 2ML-NaCl island where the interface state scattering is stronger than on 3ML for a better visualization of the scattering. The conclusions regarding the charged character of the different species on 2 or 3ML NaCl remain however the same (see Supplementary Fig. 8). The insets show topographic STM images (3.7 × 3.7 nm^2^) recorded simultaneously that reveal dark areas around HPc^−^ and Pc^2−^, characteristic of charged molecules on NaCl^35^. **b**, **f**, **j** d*I*/d*V* spectra recorded on (**b**) H_2_Pc, (**f**) HPc^−^, and (**j**) Pc^2−^ on 3ML of NaCl. **c**, **g**, **k** DFT calculations of the electronic structure of the frontier orbitals of (**c**) H_2_Pc, (**g**) HPc^−^, and (**k**) Pc^2−^ together with their iso-surface representations and their electronic occupation. **d**, **h**, **l** calculated images of the electrostatic field for (**d**) H_2_Pc, (**h**) HPc^−^, and (**l**) Pc^2−^. The potential is displayed 0.21 nm above the plane of the molecule. The NaCl layer is not included in the calculation. Source data are provided as a Source Data file.

Single and double STM-induced removal of central hydrogens of porphyrin and phthalocyanine molecules were reported in several prior works^24,29,31–33^. In some cases, it was assumed that only the proton was removed^29,31,33^ while some other works using partially decoupled molecules^24,32^ concluded on a full dehydrogenation (proton and electron). In the latter case, the HPc molecule is in a neutral state with an unpaired electron in the *π*-orbital, which strongly affects its electronic properties. The former, which has not been reported so far for decoupled molecules, should leave the molecule in a negatively charged state. Characterizing the charged state of the molecules may therefore unravel the exact nature of the STM-induced chemical reaction: dehydrogenation or deprotonation. On 2 monolayer (ML) NaCl-covered (111) noble metal surfaces, charged atoms, and molecules were shown to scatter the two-dimensional surface-state localised at the metal-salt interface (also called interface state) while neutral species do not^34,35^. In Fig. 2a, e, and i, we display differential conductance images (i.e., constant current d*I*/d*V* maps) recorded at a DC bias of 400 mV—some 200 meV above the onset of the interface state—for the same molecule prior to (a) and after the first (e) and second (i) voltage ramp procedure (the topography is shown in the inset). These images reveal circular standing waves— which become even more apparent in difference images (see Supplementary Fig. 4)—around the modified molecules that are absent for H_2_Pc. This indicates that, on NaCl, only the protons are removed from the H_2_Pc molecules ending up in singly (HPc^−^) and doubly (Pc^2−^) negative charged species. Note that the molecule occupies the same adsorption site prior to and after the deprotonation procedures (see Supplementary Fig. 5). In Fig. 2b, f and j, we first evaluate how these localised charges affect the electronic structure of the molecules by recording differential conductance (d*I*/d*V*) spectra for the three compounds. These spectra reveal similar HOMO–LUMO gaps (≈2.7 eV), as well as rigid shifts of the frontier orbitals to higher energies (+0.94 eV for HPc^−^ and +1.05 eV for Pc^2−^). For more information on the interface states and scattering of charged species, as well as on the rigidity of the electronic gap of the molecules, please refer to Supplementary Figs. 4–7. These observations further support the deprotonation mechanism, as a dehydrogenation would lead to a splitting of the HOMO into a singly occupied and a singly unoccupied molecular orbital^24^, or a shift of the original HOMO above the Fermi level^32^. The d*I*/d*V* spectra in Fig. 2b, f, j rather indicate that the *π*-orbitals are unaffected upon deprotonation, an observation that is backed-up by DFT calculations of the electronic structure of the three species (Fig. 2c, g, k), see Methods for details. In contrast, these calculations reveal large modifications of some *σ*-orbitals, originally involved in the N-H bond. For example, the orbital labeled *σ*-HOMO in Fig. 2g and k, located 1.7 eV below the *π*-HOMO in the calculation of H_2_Pc, appears 0.3 eV below and 0.4 eV above the *π*-HOMO for HPc^−^ and Pc^2−^, respectively. Altogether, these observations suggest that the excess negative charges do not localise in the frontier *π*-orbitals but in the *σ*-orbitals originally involved in the N–H bonds. In fact, similar deprotonation effects were reported for porphycene compounds which were identified as *σ*-type anions and dianions^36^. To better identify *where* the excess charges are located on the HPc^−^ and Pc^2−^ chromophores, we represent in Fig. 2d, h, and l the total electrostatic potential generated jointly by the nuclei and the distributed electron density of the singlet ground state of the molecule. The potential is displayed in a horizontal plane 0.21 nm above the molecule. The absence of clear contrast in the calculated image of H_2_Pc confirms the neutral nature of the molecule. For HPc^−^ and Pc^2−^, these calculated images reveal an overall negative potential on the molecules, that is of maximum amplitude at the center of the chromophores where the protons have been removed. This indicates that the charges left over after deprotonation remain close to their original positions, strongly affecting the *σ*-orbitals originally involved in the N–H bond but preserving the *π*-structure of the chromophore. The rigid shift of the frontier orbitals observed in Fig. 2f, j can therefore be associated to a change in the molecular ionization energy due to the electric field generated by the excess inner charges. For more information about calculations of the electronic structure and total potential, please refer to “Methods”.

### Internal Stark-shift of the molecular fluorescence lines

**Fig. 3:**
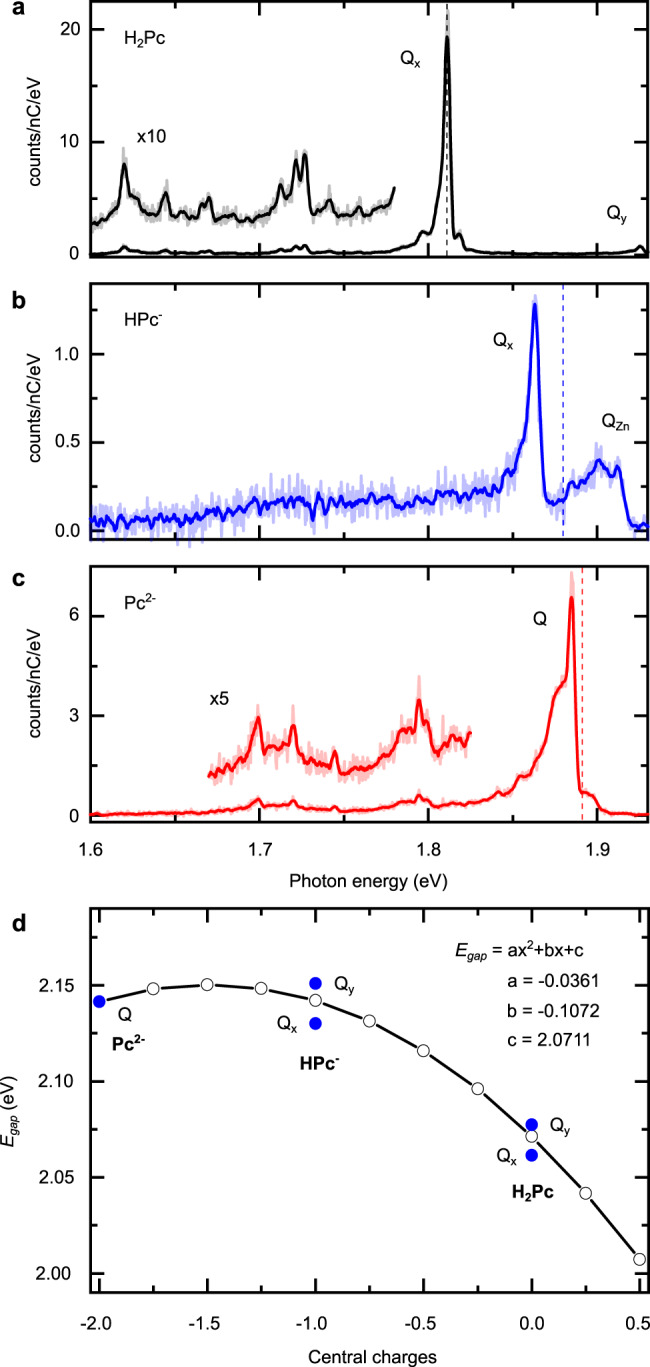
ISE in fluorescence spectra of neutral and charged phthalocyanines. STML spectra (acquisition time *t* = 120 s) of (**a**) H_2_Pc (*I* = 100 pA, *V* = −2.5 V), (**b**) HPc^−^ (*I* = 60 pA, *V* = −2.5 V), and (**c**) a Pc^2−^ (*I* = 100 pA, *V* = 2.5 V) on 3ML NaCl/Ag(111). The spectrum of HPc^−^ was obtained by resonant energy transfer from a neighboring ZnPc. The vertical dashed lines correspond to TD-DFT calculations of the S_0_ → S_1_ absorption energies for H_2_Pc, HPc^−^, and Pc^2−^, rigidly shifted by −250 meV to fit the experiment. This shift can be rationalized by considering that the dynamical screening linked to the presence of the NaCl/Ag(111) substrate and of the STM tip are not accounted for by our theoretical approach. While this prevents a quantitative comparison between experimental and theoretical energies, this procedure shows that theory accurately predicts the energy shift upon deprotonation. **d** Comparison between TD-DFT absorption energies calculated for H_2_Pc, HPc^−^, and Pc^2−^ (blue dots) and TD-DFT absorption energies calculated for a Pc^2−^ as a function of partial positive charges added artificially in its center (white dots). The line through the white dots is a parabolic fit with the parameters given in inset. Source data are provided as a Source Data file.

We now discuss the photonic properties of the three chromophores and investigate the effect of one or two central charges on the fluorescence of the phthalocyanine molecule. Figure 3 displays the STM-induced fluorescence spectra of H_2_Pc, HPc^−^ and Pc^2−^. The spectrum of H_2_Pc, excited at a negative bias of *V* = −2.5 V, is composed of two purely electronic contributions named Q_*x*_ and Q_*y*_ appearing respectively at ≈1.81 and ≈1.93 eV^26,27,37,38^. Q_*x*_ (Q_*y*_) is defined as the low (high) energy spectral contribution, which, for H_2_Pc, is associated to a dipole oriented along (perpendicular to) the axis formed by the two inner hydrogen atoms. Q_*y*_ usually appears much weaker than the lower energy Q_*x*_ contribution, reflecting fast non-radiative decay channels between the excited states^39^. Peaks of low intensities are also observed on the low energy side of the spectrum that reflect vibronic transitions characteristic of the H_2_Pc molecule^27,40^. Independently of the used polarity, HPc^−^ does not emit when directly excited by the STM tip, a behavior that will be discussed elsewhere but that we associate to the low absolute energies of both HOMO and LUMO that prevent the excitation of the molecule by tunneling electrons^41^. However, the fluorescence of HPc^−^ can be recovered through excitonic energy transfer^37,42^ from a higher energy gap molecule, here ZnPc, positioned in direct contact to the “dark” HPc^−^ (Supplementary Fig. 9). Hence, an energy-transfer mediated excitation enables probing the fluorescence of otherwise “dark” molecules, a strategy that may be used with other “dark” chromophores in STML experiments. This spectrum exhibits two electronic contributions; the high energy one can be associated to the Q_*Zn*_ emission line of the ZnPc donor^40,43^, whereas the Q_*x*_ fluorescence line of HPc^−^ is at ≈1.86 eV, some 50 meV above the Q_*x*_ peak of H_2_Pc. Note that according to our TD-DFT calculations, the low energy transition (Q_*x*_) of HPc^−^ is oriented along the molecular axis that does not contain the hydrogen atom. The Q_*y*_ contribution of HPc^−^ cannot be identified in the spectrum (Fig. 3b), probably because it is at a higher energy than the Q_*Zn*_ contribution of the ZnPc donor. For Pc^2−^, Fig. 3c shows that it can only be excited at positive voltage (*V* = 2.5 eV) with an emission line at ≈1.88 eV. Similar to metal phthalocyanines^37,40,43,44^, only a single emission line is observed in this spectrum, reflecting the D4h symmetry of the doubly deprotonated molecule and the associated degeneracy of the two first emission contributions.

In summary, one notices that moving from H_2_Pc to Pc^2−^, the transition is blue-shifted by 50 meV upon the first deprotonation, and by an additional 20 meV upon the second one. These shifts may originate from several physical effects. Due to the presence of the STM tip generating external static and dynamic electrical fields, Stark and photonic Lamb effects may contribute to the observed energy shifts^18,45–48^. However, recent STML and photoluminescence works demonstrated that these effects may at best lead to few meV shifts of the emission maxima^18,48^, and cannot explain the large blue shifts reported in Fig. 3b and c.

The interaction between the indirectly excited HPc^−^ molecule with the donor molecule of ZnPc can also result in small energy shifts^42^ of the HPc^−^ emission line, but which again are negligible compared to the shifts in Fig. 3. Similarly, we theoretically ruled out the impact of the static screening of the NaCl substrate on the energy shifts by performing DFT and TD-DFT calculations^44^ (discussed in detail in Supplementary Note 2). On the other hand, charged chromophores as *π*-type phthalocyanine anions and cations discussed in previous reports^21–23,49^ are systematically characterized by a strongly red-shifted emission (≈400 meV) compared to neutral compounds, reflecting important modifications of the *π*-orbitals involved in the optical transition. Similarly, if one assumes neutral dehydrogenated compounds, one is left with one unpaired electron in the HOMO (HPc) or with an empty HOMO (Pc)^24,32^. The fluorescence spectra of HPc and Pc should therefore reflect those of a H_2_Pc *π*-type cation (H_2_Pc^+^) and a H_2_Pc *π*-type dication (H_2_Pc^2+^) with whom they share the same electronic structure. Fluorescence spectra of HPc and Pc should therefore display emission lines red-shifted by roughly 400 meV with respect to the Q_*x*_ of H_2_Pc; this is inconsistent with the spectra of Fig. 3. In contrast, our DFT calculations of the deprotonated compounds, HPc^−^ and Pc^2−^, reveal an unchanged occupancy of the *π*-orbitals compared to H_2_Pc, explaining why the fluorescence characteristics of these three iso-electronic compounds do not change drastically. The observed blue shifts are well reproduced by the TD-DFT simulations of HPc^−^ and Pc^2−^ (assuming a systematic shift of the theoretical data to account for the specific environment of the chromophores, see Fig. 3 and “Methods” for details). These blue-shifts may either find their origin in tiny structural reorganisations of the molecule upon deprotonation, or in the electric field generated by the excess *σ*-electrons.

To address this issue, we considered the fully symmetric Pc^2−^ molecule and progressively neutralized it by artificially adding partial positive charges in its center, assuming an unchanged geometry. We then calculated the evolution of the optical gap as a function of the molecular charge (Fig. 3d). These simulations show that the optical gaps of HPc^−^ and H_2_Pc scale extremely well with those of Pc^2−^ with one and two central positive charges, respectively, eventually demonstrating that the observed shifts are due to the Stark effect generated by the internal charges.

Assuming such an ISE, it may appear surprising to observe a much smaller relative shift (+20 meV) upon removal of the second proton compared to the first deprotonation (+50 meV). This phenomenon is explained by the data of Fig. 3d, where a parabolic dependency of the optical gap on the central charge is observed, and where the singly and doubly charged chromophores are at either side of the apex of the parabola, leading to very close optical gaps. In the usual case of a chromophore with non-degenerated electronic states placed in a chiefly homogeneous external electrical field, the linear and quadratic Stark effects reflect the respective changes in the permanent dipole moment, ${{\Delta }}\vec{\mu }$, and in the polarizability, Δ*α*, experienced by the ground and excited states of the chromophore^25^. As the H_2_Pc ground and excited states do not exhibit permanent dipole moments (${\vec{\mu }}_{{S}_{0}}$ = ${\vec{\mu }}_{{S}_{1}}$ = $\vec{0}$), the presence of a non vanishing linear term (see inset Fig. 3d) may be surprising. This can be elucidated by accounting for the specific geometry of our system where central charges generate a strongly non-homogeneous electric field at the scale of the chromophore, eventually resulting in a linear contribution to the Stark shift of the spectral line (see a detailed perturbation model in Supplementary Note 3). This behavior is therefore characteristic of the close proximity between a chromophore and a point source of electric field. Clearly evidenced in our model system, this “local” effect is inherent to any ISE configuration, including the most complex biological ones, and constitutes the main difference with the usual Stark effect generated by an external electric field.

### Vibronic spectra of the deprotonated molecules

**Fig. 4:**
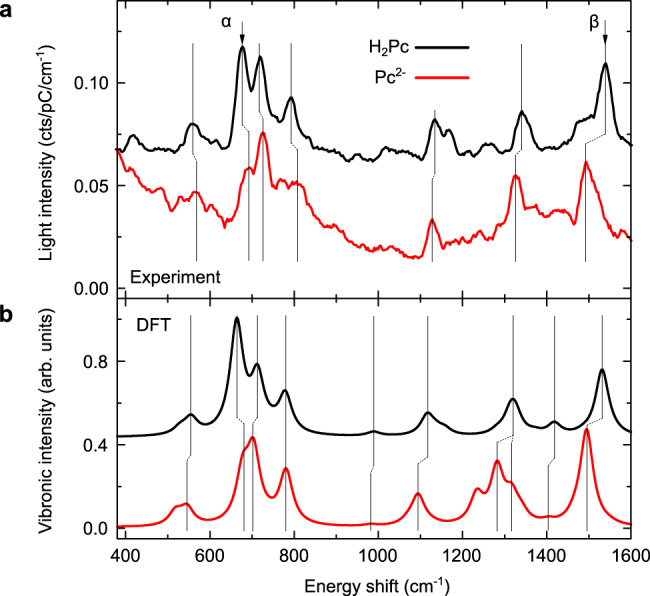
Effect of the deprotonation on the vibronic signature of the phtahlocyanine molecule. **a** STML vibronic spectra (*I* = 100 pA, acquisition time *t* = 120 s) of H_2_Pc (*V* = −2.5 V) and Pc^2−^ (*V* = 2.5 V) molecules on 3ML of NaCl compared with (**b**) theoretical vibronic intensities calculated for the molecules in vacuum. The Q_*x*_-line of H_2_Pc and Q-line of Pc^2−^ are used as the origin of the experimental energy scale. *α* and *β* indicate vibronic modes for which the four nitrogen atoms of the pyrrole cycles stand nearly still. Source data are provided as a Source Data file.

The H_2_Pc and Pc^2−^ spectra also display several vibronic emission lines, similar to tip-enhanced Raman spectra, that can be used as accurate chemical fingerprints of the probed compounds, and which provide detailed information regarding their chemical bond structures^40^. These spectra (Fig. 4a) reveal subtle changes of the intensity and energy of several vibronic peaks between H_2_Pc and its doubly deprotonated counterpart. At this stage, however, these changes can indicate either a shift of the mode frequency or the appearance/disappearance of vibronic peaks. In Fig. 4b, we show DFT calculations of the vibronic active modes for the two compounds, which reproduce the experimental frequencies and peak intensities remarkably well. We then use atomic-coordinate displacements provided by DFT to identify the prominent modes of H_2_Pc and of Pc^2−^ and indicate with black vertical lines in Fig. 4b those having a nearly perfect one-to-one correspondence. Experimentally, while the modes below 900 cm^−1^ all shift to higher wavenumbers upon double deprotonation of H_2_Pc, those above 900 cm^−1^ display the opposite behavior. Numerically, only one of the low wavenumber modes (*α*) clearly blue shifts, while the experimental trend in the high wavenumber modes is better accounted for.

Two shifts that are well reproduced by the numerical simulations, both in amplitude and sign, are those of the strongest peak close to 700 cm^−1^ (*α* in Fig. 4) and the highest wavenumber mode around 1500 cm^−1^ (*β* in Fig. 4). For both modes, the four nitrogen atoms of the pyrrole cycles, whether protonated or not, stand nearly still as do the central protons when present (see Supplementary Fig. 10 for the real-space representation of the *α* and *β* vibrational modes). Hence, their frequency shifts can hardly be explained by an isotopic-like effect associated to deprotonation. In contrast, the two modes entail large motions of the nitrogen atoms bridging the isoindole units and of the carbon atoms they are bound to. The vibronic intensities of these modes are therefore linked to the conjugation paths along the inner rings of *π*-orbitals in both species. Their shifts must therefore be attributed to a polarization of the *π*-electron system upon removal of the inner proton. To conclude, these two modes may then be seen as undergoing pure vibrational ISE. Most of the other modes entail radial motions of the nitrogen of the isoindoles. Therefore, the respective influence of the vibrational ISE and of isotopic effects induced by the removal of the central protons cannot be disentangled.

## Discussion

Charged states of single molecules have been recently probed in a wide range of experimental schemes involving scanning probe approaches^19–23,35,49^. By simultaneously preserving the *π*-orbital structure of H_2_Pc and leaving an excess *σ*-electron within the chromophore, the deprotonation procedure reported here provides a unique opportunity to study the Stark effect generated by an internal charge on the fluorescence emission of an individual chromophore. The resulting *σ*-type anionic and dianionic molecules constitute model systems allowing us to identify the role played by the proximity between a chromophore and a point-like electrostatic field source. This proximity is responsible for the combined linear and quadratic dependency of the emission energy, a behavior that should occur in any biological systems subject to ISE. It also suggests that chromophores in STML experiments could be used as precise electrostatic sensors of their nanometer-scale environment. Eventually, this work establishes a new biomimetic strategy, based on the control of the local electrostatic environment, to tune and optimise future artificial molecular optoelectronic devices.

## Methods

### Experiment

The experiments are performed in ultra-high vacuum at low temperature (≈4.5 K) with an Omicron STM that is combined with an optical set-up adapted to detect light emitted at the STM tip-sample junction. The emitted photons are collected with a lens located on the STM head and then redirected out of the chamber through optical viewports. The light is then focused on an optical fiber coupled to a spectrograph itself connected to a low-noise liquid nitrogen-cooled CCD camera. The spectral resolution of the setup is better than 1 nm. Further details regarding the optical detection setup can be found in the Supplementary Materials of ref. ^50^.

The STM tips are prepared by electrochemical etching of a tungsten wire in a NaOH solution. The tips are then sputtered with argon ions and annealed under UHV. To optimise their plasmonic response, the tips are eventually indented in clean Ag(111) to cover them with silver. The Ag(111) substrate is cleaned by simultaneous argon-ion sputtering and annealing. After this cleaning procedure, NaCl has evaporated on the Ag(111) substrate maintained at room temperature. Post-annealing at ≈370 K is performed to induce NaCl surface reorganization in bi- and tri-layers. Eventually, the 2–3ML NaCl/Ag(111) sample is introduced into the STM chamber and cooled down to ≈4.5 K. H_2_Pc and ZnPc molecules are then sublimed in very small quantities on the cold sample from a powder located in a quartz crucible. ZnPc–HPc^−^ molecular dimers on NaCl are obtained by STM tip manipulation of ZnPc. To this end, the STM tip is first positioned at the edge of a ZnPc molecule at a bias *V* = +2.5 V. In a second step, the tip-molecule distance is slowly reduced until a jump of current occurs, indicating a motion of the molecule. The procedure is reproduced until the desired structure in obtained. The d*I*/d*V* maps of Fig. 2 in the main paper are recorded in constant current mode (closed feedback loop) with a voltage modulation of 50 mV at a DC voltage of 400 mV, whereas the d*I*/d*V* spectra are recorded in constant height mode (open feedback loop) with a voltage modulation of 20 mV. A modulation frequency of 740 Hz is used for these measurements.

### DFT calculation of the ground-state electronic properties

To analyze the molecular transport properties, the electronic structure of the molecule in a vacuum, and the distribution of the corresponding electron charge density in the neutral and charged species, we perform ground-state DFT calculations using the OCTOPUS code^51^. In OCTOPUS the electron density is represented on a real-space grid which does not constrain the localization of the density to predefined atomic orbitals and is therefore suitable to describe the ground-state electron densities of the charged molecules. The electron-ion interaction is modeled in the framework of the pseudopotential approximation. We use the Perdew-Zunger^52^ parametrization of the local density approximation (LDA) correlation and the Slater density functional for the LDA exchange functional^53,54^. We present further analysis of the molecular orbitals, electronic density, and comparison with experimental d*I*/d*V* images of the molecules with theory in Supplementary Figs. 1–3 and Supplementary Table 1.

### Numerical analysis of the ground-state electron densities

We extract from OCTOPUS the ground-state electron densities and calculate the total electrostatic potential *ϕ*_tot_ generated by the molecular charges to visualize the localization of the excess charge of the deprotonized molecules (as shown in Fig. 2). To that end we solve the Poisson equation:

1 $${{\Delta }}{\phi }_{{{{{{{{\rm{tot}}}}}}}}}=\frac{{\rho }_{{{{{{{{\rm{e}}}}}}}}}({{{{{{{\bf{r}}}}}}}})}{{\varepsilon }_{0}}+\frac{{\rho }_{{{{{{{{\rm{ion}}}}}}}}}({{{{{{{\bf{r}}}}}}}})}{{\varepsilon }_{0}},$$

with *ε*_0_ being the vacuum permittivity. *ρ*_e_(**r**) is the electron charge density of the valence electrons in the singlet ground state of the molecule and *ρ*_ion_(**r**) is the charge density of the positive nuclei screened by the core electrons of the respective atoms. We represent the density of the screened nuclei as a sum of Gaussian charge distributions:

2 $${\rho }_{{{{{{{{\rm{ion}}}}}}}}}({{{{{{{\bf{r}}}}}}}})=\mathop{\sum}\limits_{i}\frac{{Q}_{i}}{{\left(2\pi \right)}^{3/2}{\sigma }_{{{{{{{{\rm{ion}}}}}}}}}^{3}}\exp\left( -\frac{{\left|{{{{{{{\bf{r}}}}}}}}-{{{{{{{{\bf{R}}}}}}}}}_{i}\right|}^{2}}{2{\sigma }_{{{{{{{{\rm{ion}}}}}}}}}^{2}}\right),$$

where *Q*_*i*_ is the total charge of the screened ion *i* at position **R**_*i*_. The charge distribution has a width *σ*_ion_ = 0.05 nm. The electron charge density is extracted from the ground-state DFT calculations in the form of Gaussian cube files. The ground-state electron charge densities for H_2_Pc and HPc^−^ are shown in Supplementary Fig. 3c, d, respectively, alongside with the corresponding geometries of the molecules (H_2_Pc in Supplementary Fig. 3a and HPc^−^ in Supplementary Fig. 3b). Finally, we solve the Poisson equation [Eq. (1)] numerically on a homogeneously spaced grid by a Fourier-based method.

### TD-DFT calculations of the excited-state electronic properties

To address the excited-state properties of the molecules we perform TD-DFT calculations as implemented in the software Gaussian 09 Revision D.01^55^. The TD-DFT calculations (Fig. 3) are carried out with the B3LYP functional and the 6-311G(d,p) basis set. The convergence of 64 roots is asked for when calculating the excited singlet states at the ground state equilibrium geometries of all three compounds which are optimized using the same functional and basis set.

The dependence on a central fractional charge of the transition energy to the first excited singlet state of the doubly deprotonated Pc dianion (Fig. 3d) is calculated in the same way while keeping its equilibrium D4h geometry frozen.

### DFT calculation of the geometry of the molecules on NaCl

The geometrical structure of the molecules on NaCl is relaxed using Quantum Espresso^56^ which is a plane-wave pseudopotential DFT code suitable for the description of the extended substrate. The cubic supercell (*a* = 53.02457485  Å) included 100 Na and 100 Cl substrate atoms and the calculation is performed at the Γ point. The exchange and correlation terms are described using the local density approximation under the approach of Perdew and Zunger^52^. Projector augmented-wave pseudopotentials with core corrections are used to describe the electron-ion interaction^57^. The energy cutoff and density cutoff are set to 50 Ry and 500 Ry, respectively. A damped dynamics is used to perform the structural optimization of the system. The atoms are moved according to Newton’s equation by using a Verlet algorithm^58^. The structural optimization is stopped when two subsequent total energy evaluations differed by <10^−4^ Ry and each force component is less than 10^−3^ Ry/bohr.

### DFT and TD-DFT calculations of the molecular vibronic properties

Finally, the vibronic intensities (Fig. 4b) are calculated as the square of the sum of the Franck and Condon and Herzberg-Teller amplitudes for each relevant mode independently using Gaussian 09 Revision D.01. To this end, the results of the normal mode calculations are used to define equally spaced discrete distortions spanning the range between the classical turning points of each mode. Repeated TD-DFT calculations of the vertical transition energy and dipole moment to the lowest excited singlet are used to determine the mode displacements and the derivative of the transition dipole moments with respect to the dimensionless normal coordinate through a series of third order polynomial regressions. Since the third order terms are found to be negligible and since the changes in frequencies (from second order terms) between ground and excited states of all the modes that are identifiable in the experimental spectrum are all well below 5% and would not affect the Franck and Condon overlaps, Duschinsky rotations (mode mixing in the excited state) are ignored. The theoretical vibronic spectra presented are then calculated using the TD-DFT determined intensities by scaling the normal mode frequencies obtained analytically at the DFT level by a factor 0.96^59^ and by broadening each line by a Lorentzian 20 cm^−1^ wide at half maximum.

## Supplementary information

Supplementary information

## Data Availability

The data supporting the findings of the present study can be found in Methods or Supplementary Information. All the datasets are also available from the corresponding author upon request. Source data are provided with this paper.

## Source data

Source Data
