# Supplementary material for: Internal Stark effect of single-molecule fluorescence
Source: Nat Commun. 2022 Feb 3;13:677. doi: 10.1038/s41467-022-28241-8 (PMC8813982; doi:10.1038/s41467-022-28241-8)
Supplement: Supplementary file 1 — Supplementary information [file 41467_2022_28241_MOESM1_ESM.pdf]

Supplementary Information: Internal Stark effect of single-molecule fluorescence.

Kirill Vasilev[1], Benjamin Doppagne[1], Tomáš Neuman[1],
Anna Rosławska[1], Hervé Bulou[1], Alex Boeglin[1]
Fabrice Scheurer[1], Guillaume Schull[1*]
[1] Université de Strasbourg, CNRS, IPCMS, UMR 7504, F-67000 Strasbourg, France,

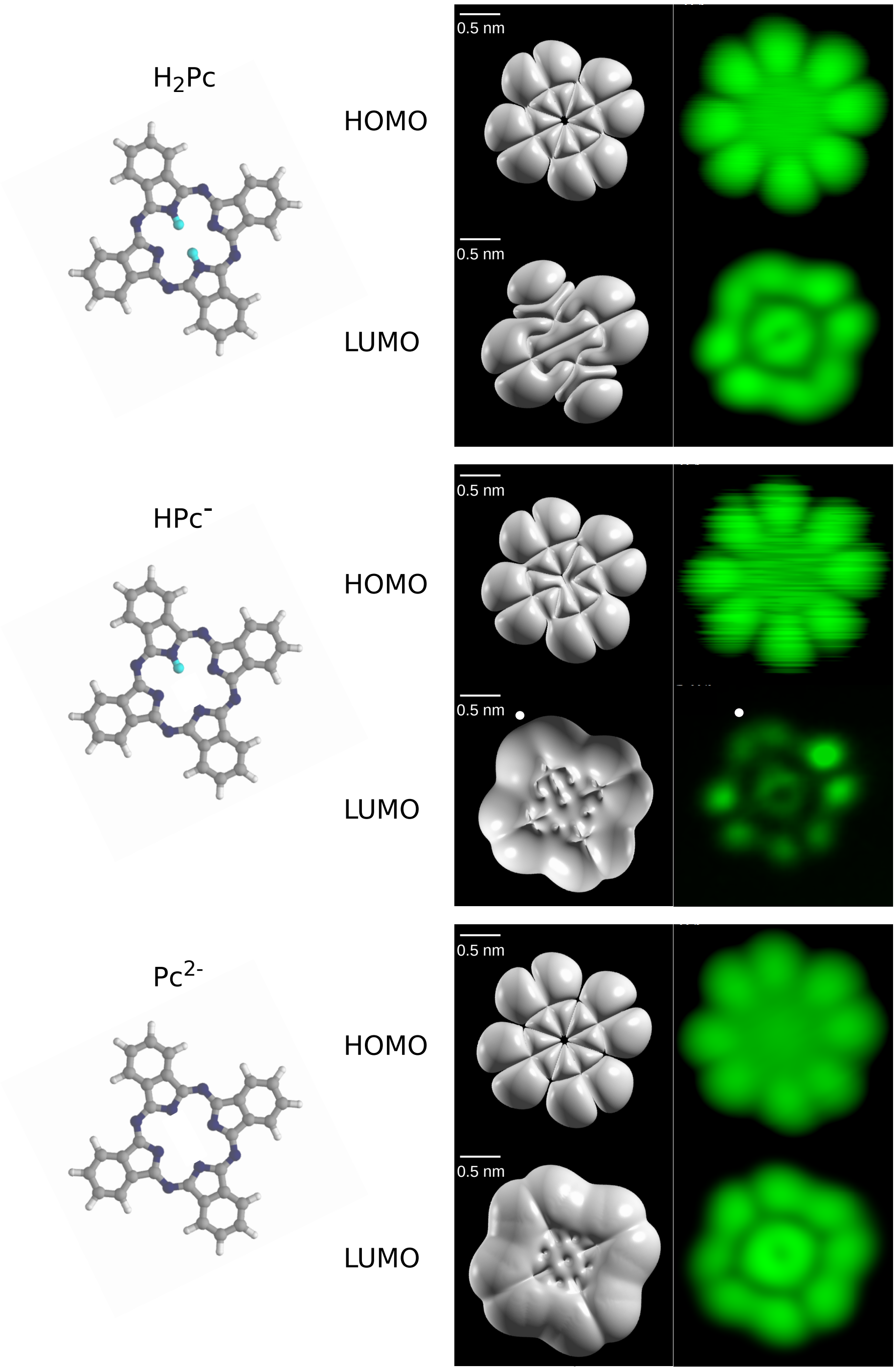

Supplementary Figure 1: **Comparison between experimental molecular orbital images and their simulations using OCTOPUS.** Left: Ball-and-stick models of H_2Pc, HPc^- and Pc^{2-} molecules. Right: (3.3×3.3 nm^2) simulated isodensity contours of Kohn–Sham orbitals (grey) and experimental STM topographic images (green) recorded at energies corresponding to the appearance of the molecular orbitals in the dI/dV spectra [for H_2Pc $V = 0.5$ V (LUMO) and V = -2.5 V (HOMO), for HPc^- $V = 1.43$ V (LUMO) and V = -1.5 V (HOMO), for and Pc^{2-} $V = 1.65$ V (LUMO) and V = -2 V (HOMO)].

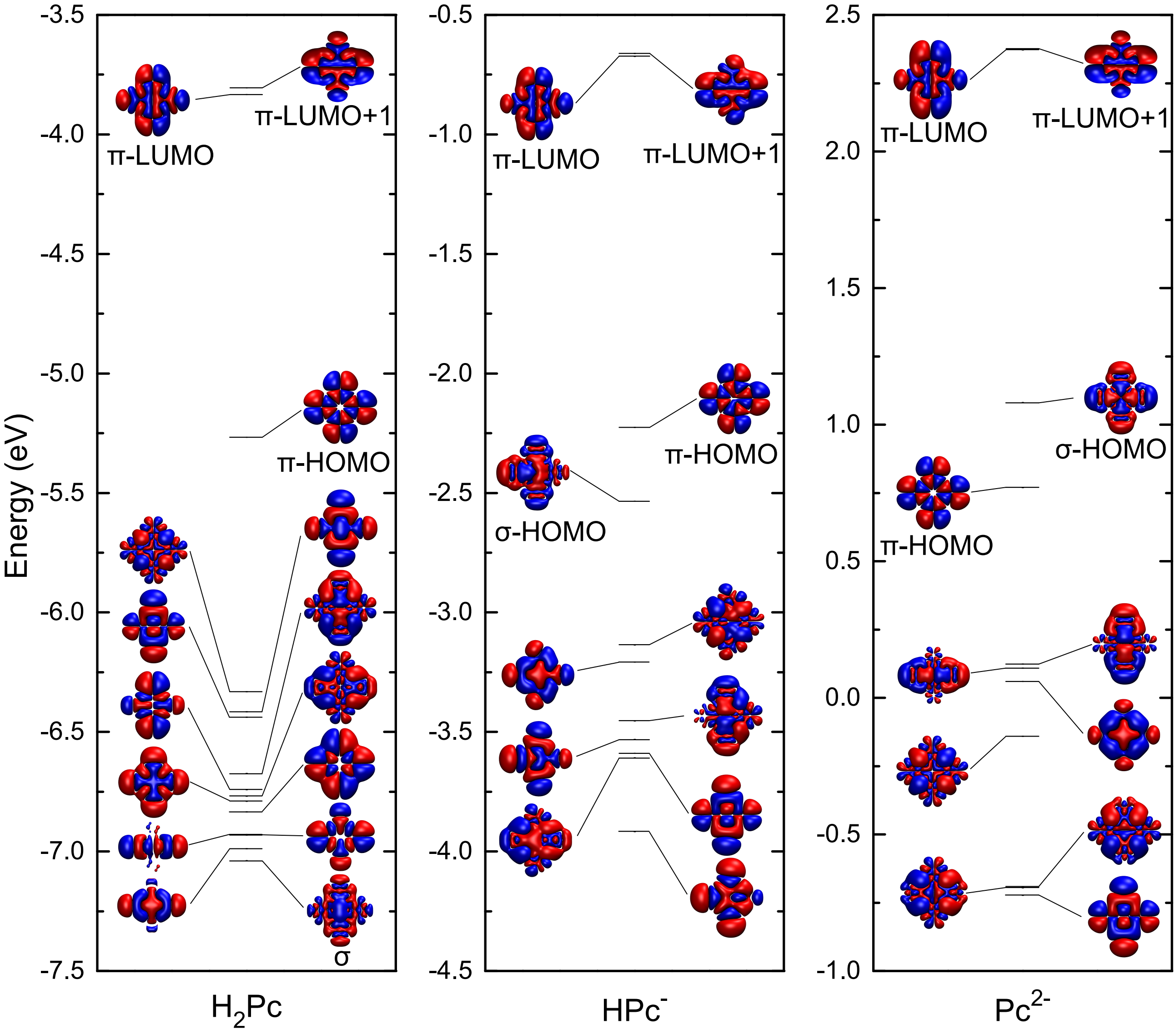

Supplementary Figure 2: **Orbitals of H_2Pc, HPc$^-$, and Pc^{2-} and their corresponding Kohn-Sham energies.** The orbitals and energies have been obtained from OCTOPUS and correspond to the data shown in Fig. 2 c,g,k, respectively. Their absolute scale corresponds to the raw output data from the calculation. The large absolute offset of the π-HOMO energies is due to the total charge of the molecule and does not take into account screening effects due to the substrate. Finally, we note that the interpretation of Kohn-Sham energies as electron affinities or ionization energies is problematic, as expected for negative ions [1].

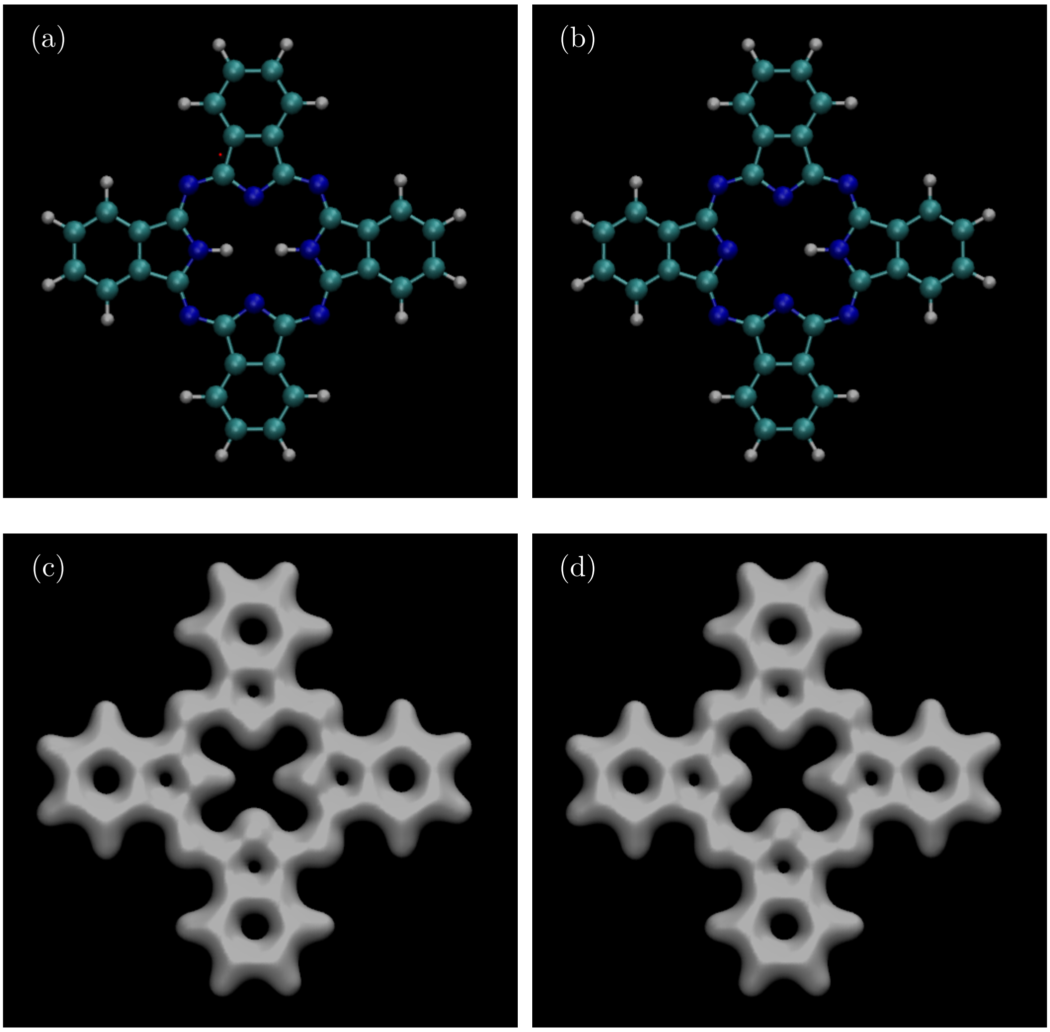

Supplementary Figure 3: **DFT calculations of geometry and charge density using OCTOPUS**. Structure of (a) H_2Pc and (b) HPc^-; (c) and (d) corresponding ground state total electronic densities.

Supplementary Table 1: **Kohn-Sham energies of orbitals extracted from OCTOPUS** (shown in Supplementary Fig. 2).

Number of orbital	H_2Pc	HPc^-	Pc^{2-}
LUMO+1	-3.805389 eV (π)	-0.661679 eV (π)	2.375192 eV (π)
LUMO	-3.833108 eV (π)	-0.673323 eV (π)	2.371648 eV (π)
HOMO	-5.267512 eV (π)	-2.225501 eV (π)	1.081084 eV (σ)
HOMO-1	-6.331576 eV (σ)	-2.534222 eV (σ)	0.769765 eV (π)
HOMO-2	-6.416079 eV (π)	-3.135890 eV (σ)	0.123006 eV (σ)
HOMO-3	-6.438473 eV (π)	-3.207126 eV (π)	0.108883 eV (σ)
HOMO-4	-6.673584 eV (σ)	-3.453955 eV (σ)	0.059415 eV (π)
HOMO-5	-6.741199 eV (π)	-3.530947 eV (π)	-0.140933 eV (σ)
HOMO-6	-6.768270 eV (σ)	-3.589132 eV (π)	-0.691981 eV (σ)
HOMO-7	-6.790280 eV (π)	-3.608158 eV (σ)	-0.696350 eV (σ)
HOMO-8	-6.834313 eV (π)	-3.916768 eV (π)	-0.723128 eV (π)
HOMO-9	-6.929237 eV (π)	-	-
HOMO-10	-6.931303 eV (π)	-	-
HOMO-11	-6.989038 eV (π)	-	-
HOMO-12	-7.038707 eV (σ)	-	-

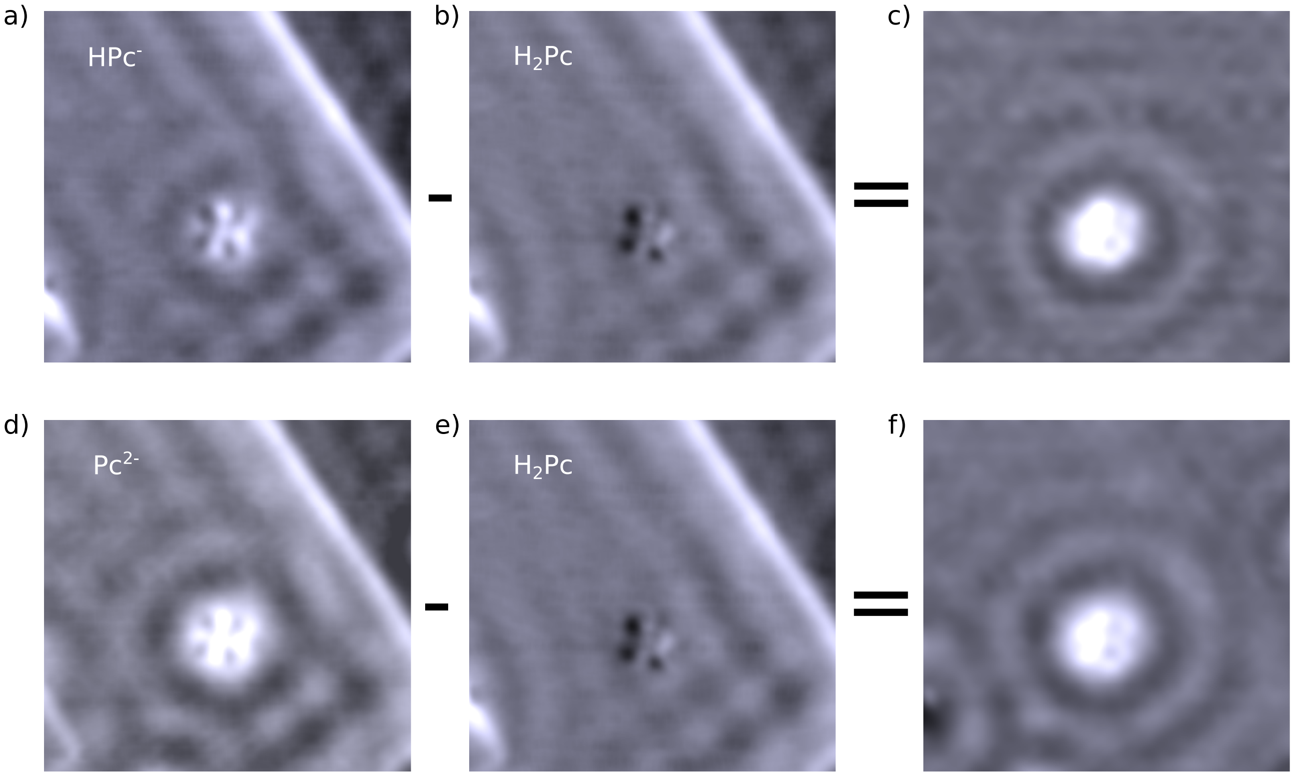

Supplementary Figure 4: **Evidencing the interface state scattering by charged molecules.** dI/dV maps (I = 30 pA, V = 400 mV, 11.0 × 10.5 nm^2) of (b, e) H_2Pc, (a) HPc^-, and (d) Pc^{2-} on 2ML NaCl/Ag(111). Subtracting the image of the neutral species from the one of the deprotonated molecules removes the effects of other scatterers (steps, defects...) and singularizes the changes occuring upon deprotonation. The resulting images (c) and (f) reveal concentric circles around the deprotonated molecules, demonstrating a change of the molecular charge state [2].

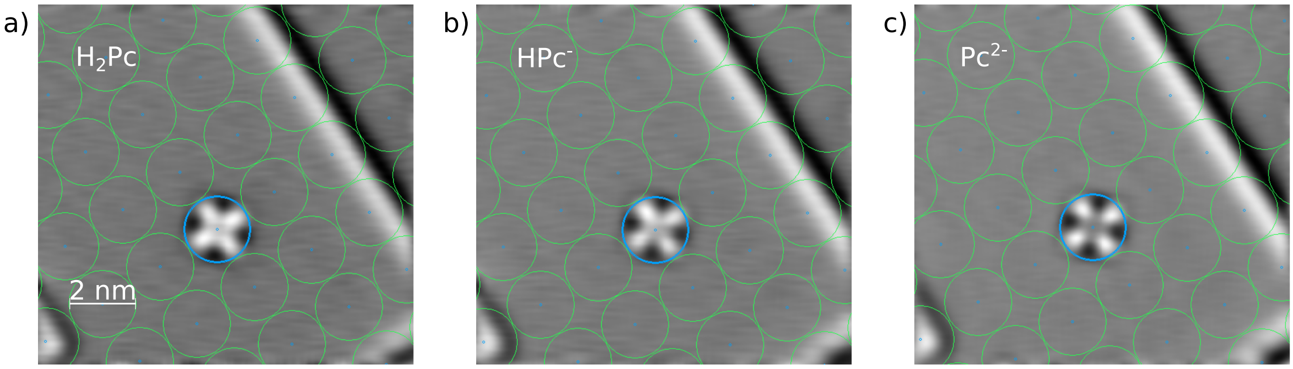

Supplementary Figure 5: **Topography images of H_2Pc, HPc^{-}, and Pc^{2-} covered with an artificial lattice.** An artificial square lattice, consisting of circles of 2 nm in diameter, is plotted on the experimental topography images (I = 30 pA, V = 400 mV, 11.0 × 10.5 nm^2) of (a) H_2Pc, (b) HPc^{-}, and (c) Pc^{2-} on 2ML of NaCl, to evaluate the position of the molecule with respect to the NaCl lattice upon deprotonation. These data indicate that H_2Pc, HPc^{-}, and Pc^{2-} occupy the same adsorption site (see Supplementary Note 2 for the detailed geometries).

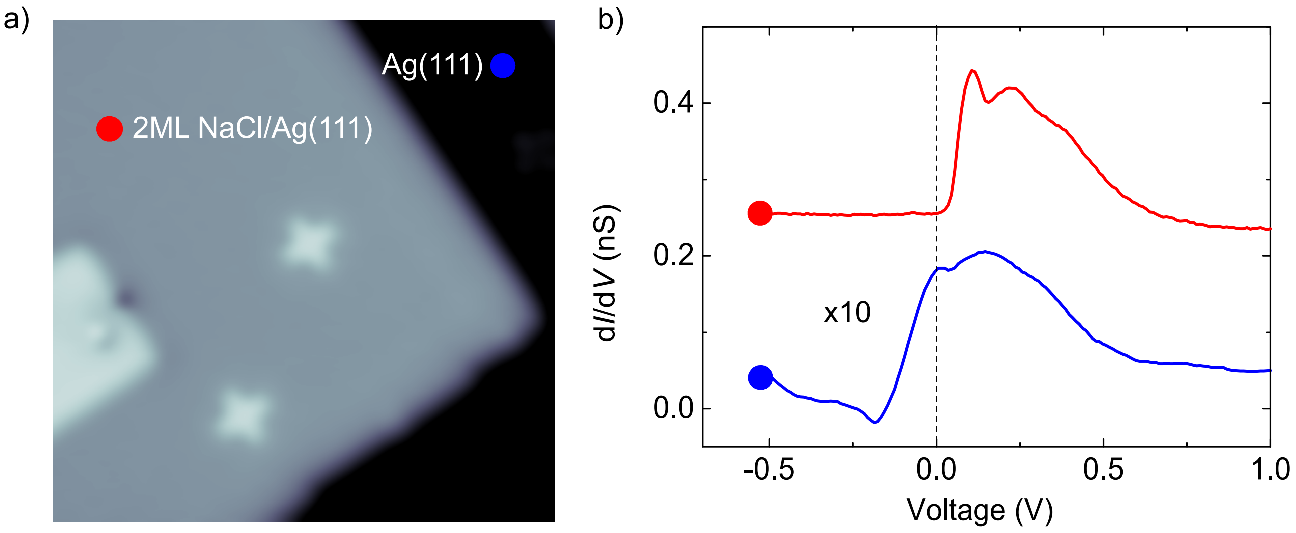

Supplementary Figure 6: **Ag(111) and NaCl/Ag(111) interface states.** (a) STM image (I = 30 pA, V = 400 mV, 15 $\times$ 15 nm^2) of a 2ML NaCl island deposited onto a Ag(111) crystal surface with two H_2Pc molecules on top. (b) dI/dV conductance spectra acquired at positions marked by color dots in (a) revealing the Ag(111) surface state onset ($\approx$ -80 meV, blue spectrum) and NaCl/Ag(111) interface state onset ($\approx$ 100 meV, red spectrum).

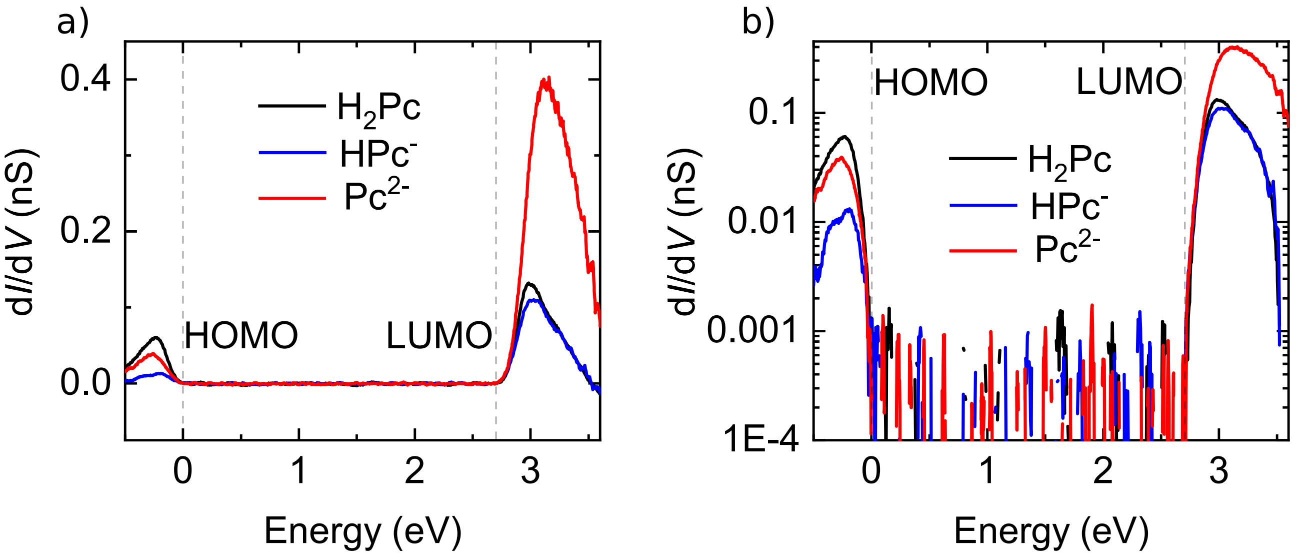

Supplementary Figure 7: **Electronic gap for the three species.** (a) dI/dV spectra of H_2Pc, HPc^- and Pc^{2-}, where the onset energy of the HOMO is taken as a reference of the energy-axis. (b) Same spectra in a logarithmic scale. These data reveal no measurable variation of the HOMO-LUMO gap upon deprotonation (≈2.7 eV), confirming the iso-electronic nature of the three compounds.

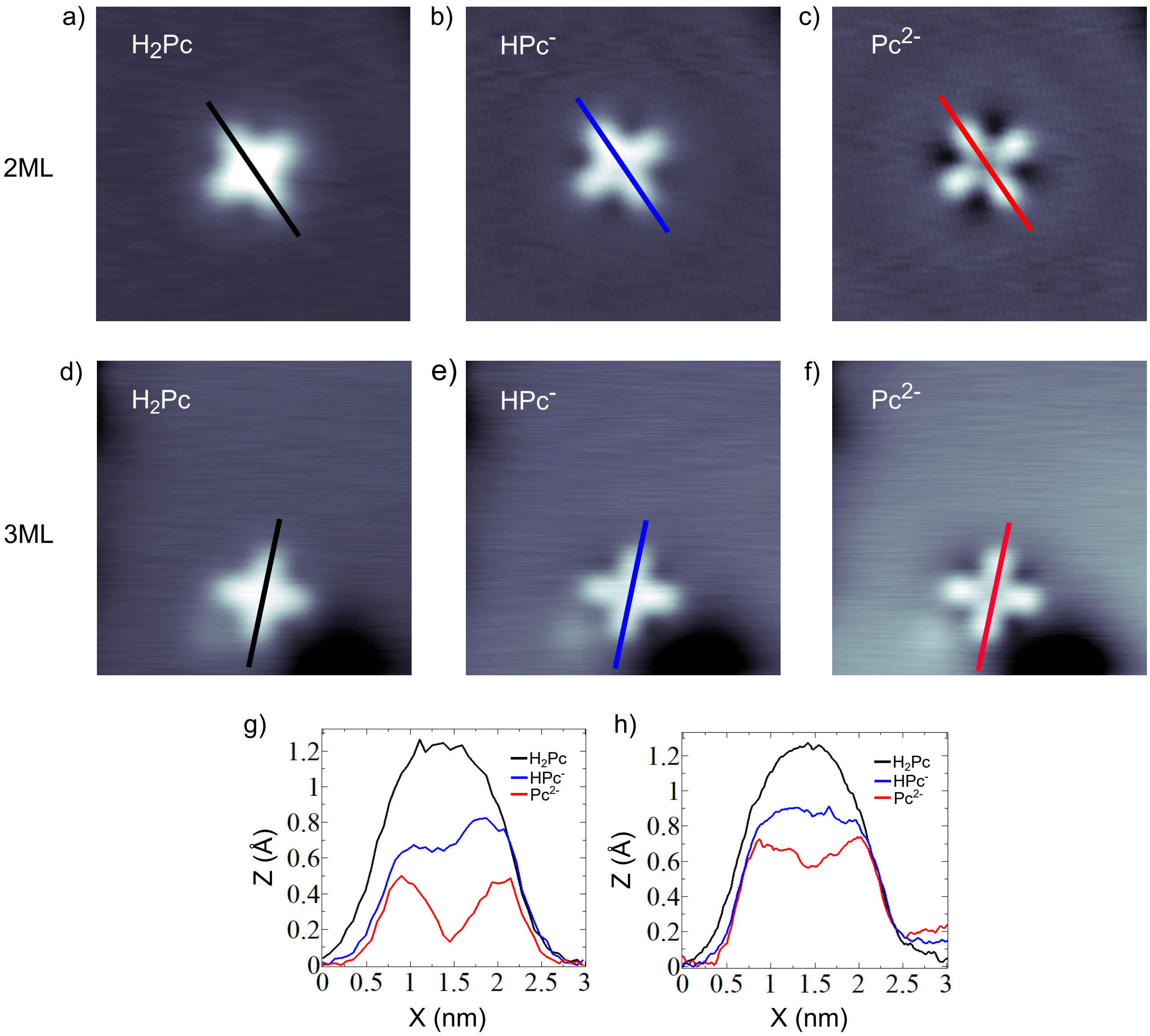

Supplementary Figure 8: **STM topography images of H_2Pc, HPc^-, and Pc^{2-}on 2 and 3 NaCl layers.** Images on 2ML of NaCl (I = 30 pA, V = 400 mV, 6.6 × 6.6 nm^2; (a) H_2Pc, (b) HPc^-, and (c) Pc^{2-}) and on 3ML (I = 10 pA, V = 400 mV, 6.6 × 6.6 nm^2; (d) H_2Pc, (e) HPc^-, and (f) Pc^{2-}). These images were acquired to study the effect of the NaCl thickness on the charge state of the deprotonated molecules. The deprotonated molecules reveal a reduction of the apparent height (see line-scans in (g), (h)) on the molecule on both 2 ML (g) and 3 ML (h) of NaCl, as well as an increased dark area around the molecules when the number of charges in the molecule increases. These two aspects are characteristic of charged molecules [2] and indicate very similar properties for the deprotonated molecules on 2 and 3ML.

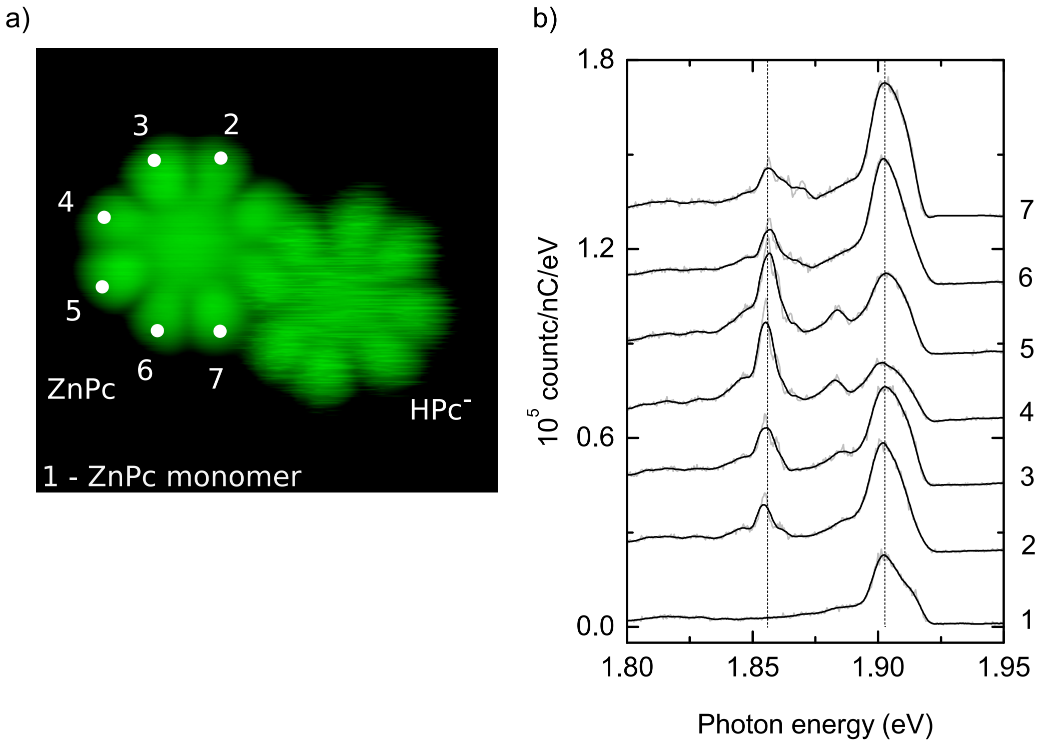

Supplementary Figure 9: **Indirect excitation of a HPc$^-$ molecule via ZnPc in a dimer configuration**. (a) STM image (I = 10 pA, V = -2.5 V, 4.7 × 4.5 nm^2) of a ZnPc – HPc$^-$ dimer (see Materials and Methods for details regarding the manipulation procedure). (b) STML spectra (right, V = -2.5 V, I = 60 pA, acquisition time = 120 s) acquired for the tip located at the positions marked in the STM image in (a). Based on spectrum (1) acquired on a ZnPc monomer, the peak at 1.9 eV can be assigned to the fluorescence of ZnPc. The peak at ≈ 1.86 eV is assigned to the HPc$^-$ transition. Note that the intensities of the ZnPc and HPc$^-$ fluorescence peaks vary with tip position, indicating more or less efficient energy transfer.

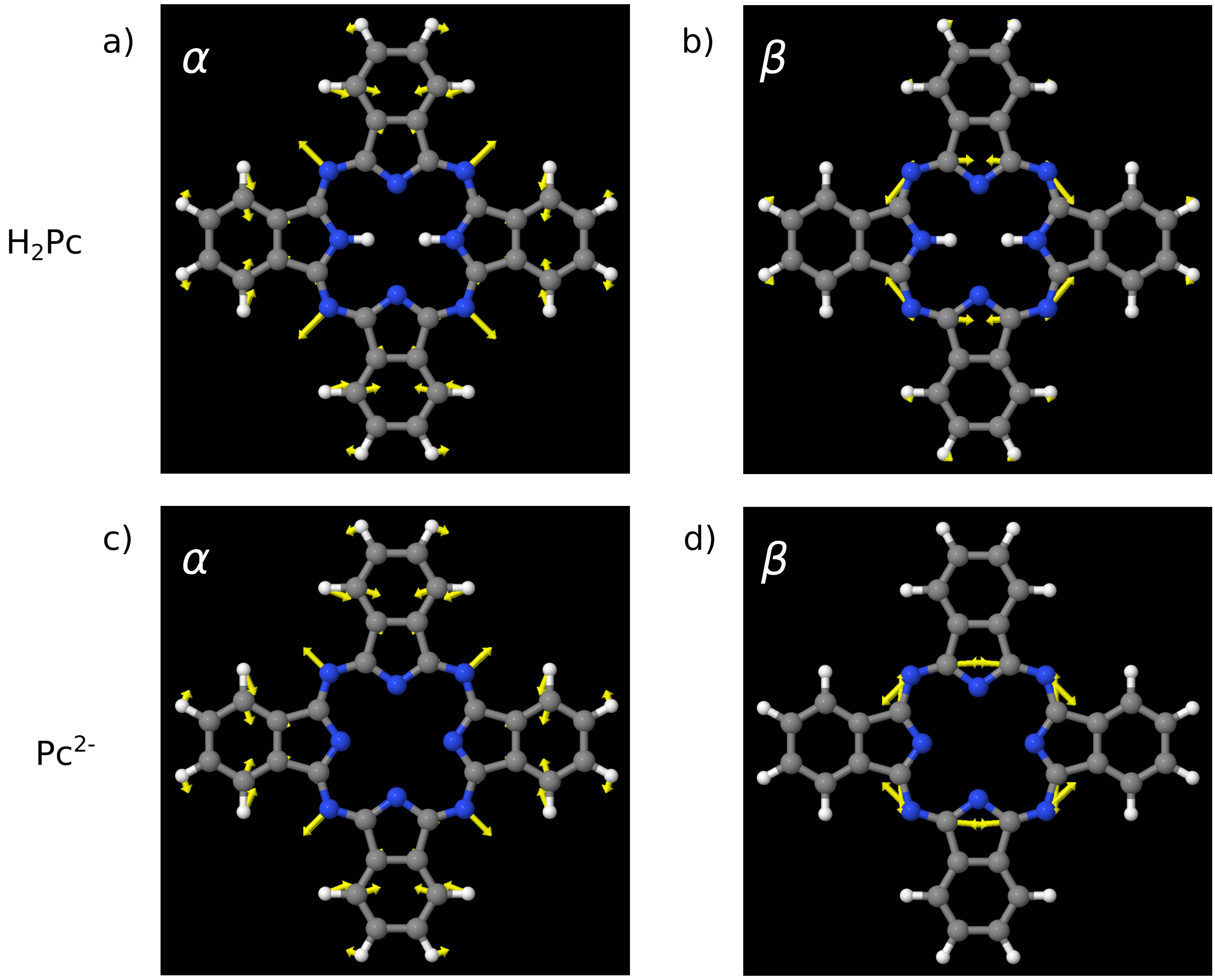

Supplementary Figure 10: **Ball-and-stick representation of the vibrational modes obtained from Gaussian.** Modes of interest of H_2Pc and Pc^{2-} species labeled as α [(a) and (c)] and β [(b) and (d)], that are specifically mentioned in Fig. 4 of the manuscript, were simulated to see the exact atom movements. Yellow arrows indicate movements of atoms, that take part in those particular vibrational modes. This clearly indicates that the four central N atoms, bonded with H atoms participating in deprotonation, are not moving.

Supplementary Note 1.

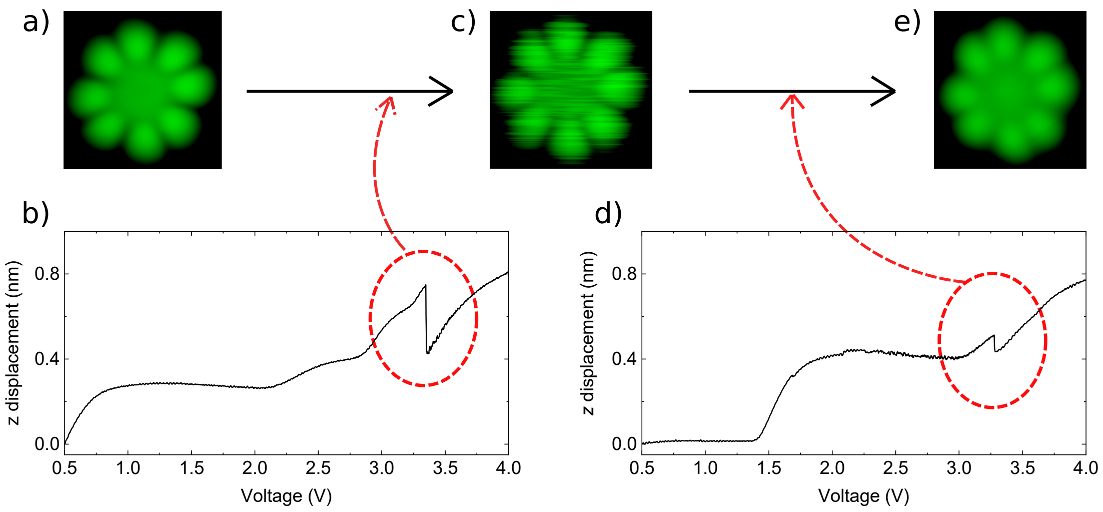

Supplementary Figure 11: **Deprotonation procedure**. (a, c, e) STM topographic images (2.7 $\times$ 2.7 nm^2) of (a) a H_2Pc molecule (I = 10 pA, V = -2.5 V) successively deprotonated to form (b) HPc^- (I = 10 pA, V = -1.5 V) and (c) Pc^{2-} (I = 10 pA, V = -2 V). (b, d) $z(V)$ plots recorded for the STM tip located at the center of (b) H_2Pc and (c) HPc^-. The dashed circles indicate abrupt z changes associated to the deprotonation events.

For the deprotonation procedure, the STM tip is positioned directly on top of the center of a H_2Pc molecule on NaCl. The bias voltage is then scanned from V = 0.5 V to 4 V at a constant current of 10 pA, while monitoring the "z" position of the piezo-controller. Abrupt changes of the tip-sample distance during this procedure (as those in Fig. 11) indicate modifications of the molecular structure. STM images and time traces (Supplementary Fig. 11 and Fig. 1 of the main text) recorded upon using this procedure allow us to assign these abrupt changes to the successive removal of the central protons of the H_2Pc molecule. For both H_2Pc and HPc^- the deprotonation occurs at $V \approx 3.25$ V. Deprotonation may occur at slightly larger or lower voltages in successive experiments.

Supplementary Note 2.

To verify that the spectral shifts observed for the H_2Pc, HPc^-, and Pc^{2-} molecules originate from the internal Stark effect (as discussed in the main text), and not from a modified interaction with the NaCl substrate, we study the influence of the static screening due to the NaCl substrate on the optical properties of the molecules (the energy of the lower excited state). To that end we perform a series of combined DFT and TD-DFT calculations. We consider the following four scenarios: (1) We relax the structures of the different species adsorbed on the Na site of the NaCl layer using Quantum Espresso, as shown in Supplementary Fig. 12a-c and as described in Materials and Methods. Subsequently, we perform a TD-DFT study of the vertical excitation energies of these molecules relaxed on the substrate and include the Na (Cl) atoms as external positive (negative) static point charges. The results are shown as full red dots connected by a full red line in Supplementary Fig. 13. (2) The geometries of the molecules relaxed on the substrate are used to perform TD-DFT calculations, but the NaCl substrate is not included (shown as full red diamonds connected by red dashed line in Supplementary Fig. 13) (3) We relax the molecular geometries in gas phase and in TD-DFT calculations we include the substrate as external point charges considering the geometry shown in Supplementary Fig. 12d-f. In this geometry, the molecule is 0.6 nm above the surface of the NaCl substrate. The substrate geometry for all adsorbed molecules (H_2Pc, HPc^-, and Pc^{2-}) is considered identical to the NaCl geometry obtained for H_2Pc adsorbed on NaCl in scenario 1. The results are shown as blue stars connected by a full blue line in Supplementary Fig. 13. (4) Finally we perform TD-DFT calculations of the molecules considering their geometries fully relaxed in gas phase and not considering the substrate (as presented in Fig. 3 d) of the main text). The result is presented as full blue squares connected by blue dashed line in Supplementary Fig. 13.

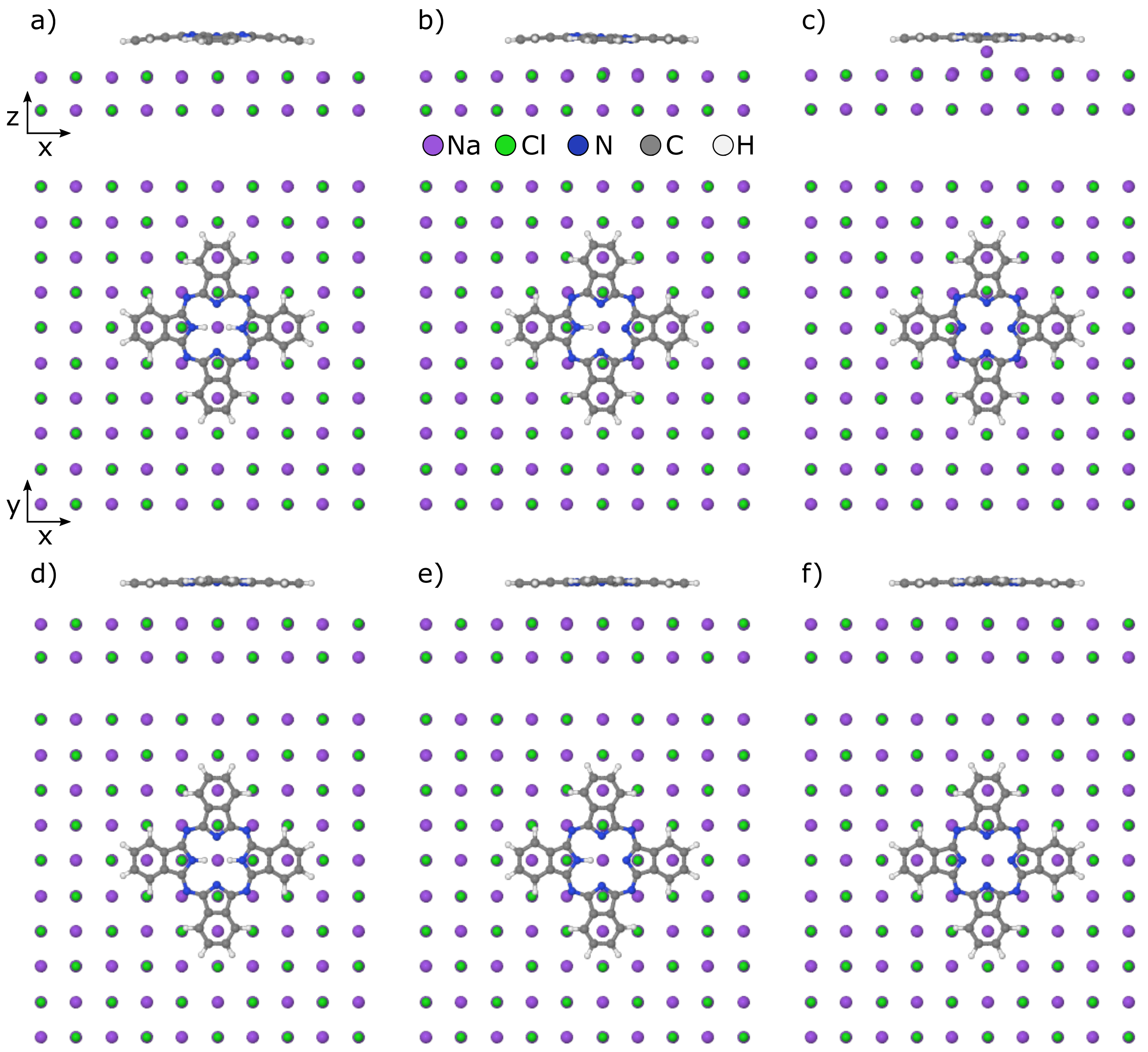

Supplementary Figure 12: **Geometry of H_2Pc, HPc^-, and Pc^{2-} on two monolayers of salt.** (a) H_2Pc, (b) HPc^-, and (c) Pc^{2-} whose geometries have been relaxed together with the top monolayer of NaCl using Quantum Espresso. (d) H_2Pc, (e) HPc^-, and (f) Pc^{2-} relaxed in the vacuum phase and inserted 0.6 nm above the top NaCl layer. The geometry of the NaCl substrate in (d)-(f) was not relaxed together with the respective molecules and is identical to the one shown in (a).

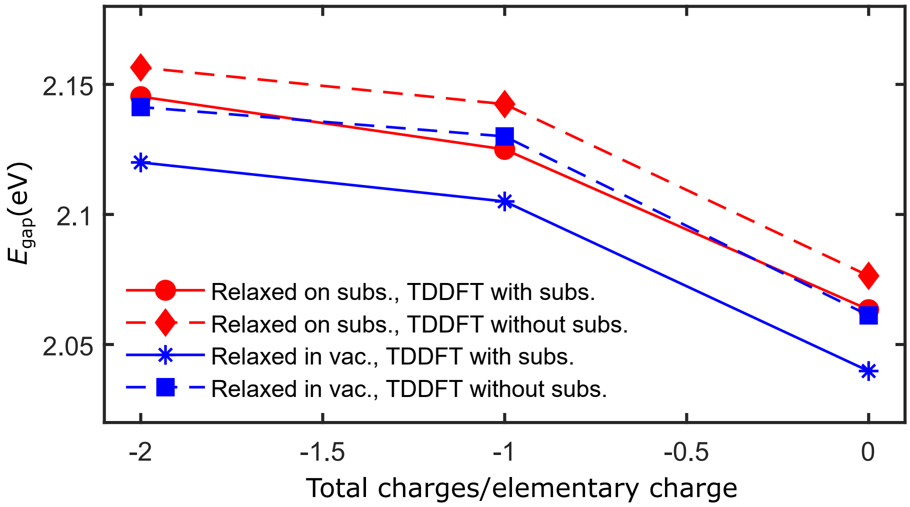

Supplementary Figure 13: **TD-DFT vertical transition energy gap (E_{gap}) of H_2Pc, HPc^-, and Pc^{2-}.** We present four different series of calculations as described in the text: Molecular geometry relaxed on the NaCl substrate, combined with TD-DFT not considering the substrate (red circles); molecular geometry relaxed on the NaCl substrate, combined with TD-DFT including the substrate (red diamonds); molecular geometry relaxed in vacuum, combined with TD-DFT including the substrate (blue stars); molecular geometry relaxed in vacuum, combined with TD-DFT not considering the substrate (blue squares). The geometry of the molecules and the salt layer for the respective situations is shown in Supplementary Fig.12. We conclude that the substrate induces a minor red shift of the vertical optical transition energy of the molecules, but does not change the phenomenology of the internal Stark effect described in this paper.

Supplementary Note 3.

In Fig. 3d of the main manuscript one notices that the Stark shift is a parabolic function of the charge appearing upon deprotonation

$$\Delta_{SS} = aZ + bZ^2 \tag{S1}$$

Here, we will rationalize this dependency by a perturbation model. Let us assume that H_0 is the time-independent Hamiltonian describing the H_2Pc molecule, having energy eigenvalues E_n^0 and eigenfunctions $|\Psi_n^0\rangle$. The Hamiltonian of the (singly or doubly) deprotonated molecule is H_1, with eigenenergies and eigenvectors E_n and $|\Psi_n\rangle$ respectively. We assume that H_1 can be written as a small perturbation to H_0: $H_1 = H_0 + V$. This assumption is supported by DFT calculations (see Materials and Methods in the main text for details), as can be drawn from Supplementary Fig. 3 which shows the ground state total electron density for H_2Pc and HPc^-. No major difference can be observed between the densities of the neutral and the charged molecule. Only a careful examination shows slight differences in the electron density close to the removed proton location (Supplementary Fig. 3c, d).

From time-independent perturbation theory [3], the molecular energy shift of the nth level of the system at the second order of the perturbation potential V reads

$$E_n = E_n^0 + \langle\Psi_n^0| V |\Psi_n^0\rangle + \sum_{k\neq n} \frac{|\langle\Psi_k^0| V |\Psi_n^0\rangle|^2}{E_n^0 - E_k^0} + \cdots , \tag{S2}$$

The energy of the lowest transition of the H_2Pc molecule is given by the difference between the energies of the two lowest lying states of the molecule, S_0^0 and S_1^0, $E_1^0 - E_0^0$. It corresponds to the quantity E_{gap} displayed in Supplementary Fig. 3d. It means that the change in energy with respect to the neutral molecule is given by

$$\Delta_{01} = (E_1 - E_0) - \left(E_1^0 - E_0^0\right). \tag{S3}$$

Using Eq. S2 one obtains

$$\Delta_{01} = \Delta_{01}^{(1)} + \Delta_{01}^{(2)}, \tag{S4}$$

where $\Delta_{01}^{(1)}$ and $\Delta_{01}^{(2)}$ are the first and second order corrections.

$$\Delta_{01}^{(1)} = \langle\Psi_1^0| V |\Psi_1^0\rangle - \langle\Psi_0^0| V |\Psi_0^0\rangle , \tag{S5}$$

$$\Delta_{01}^{(2)} = 2\frac{|\langle\Psi_0^0| V |\Psi_1^0\rangle|^2}{E_1^0 - E_0^0} + \sum_{k>1}\left(\frac{|\langle\Psi_k^0| V |\Psi_1^0\rangle|^2}{E_1^0 - E_k^0} - \frac{|\langle\Psi_k^0| V |\Psi_0^0\rangle|^2}{E_0^0 - E_k^0}\right). \tag{S6}$$

Here, the perturbation is due to the deprotonation, which is equivalent to a repulsive potential of negative point charges. Assuming that the point charges are localized at the center of the molecule, the potential (in atomic units) is

$$V(\boldsymbol{r}) = Z\frac{1}{\mid \boldsymbol{r} \mid} = Zv(\boldsymbol{r}). \tag{S7}$$

with Z the number of removed protons ($Z = 1$ for HPc^{-}, $Z = 2$ for Pc^{2-}). Replacing in Eq. S6 one obtains

$$\Delta_{01}^{(1)} = Z\left(\langle\Psi_1^0|\, v\,|\Psi_1^0\rangle - \langle\Psi_0^0|\, v\,|\Psi_0^0\rangle\right), \tag{S8}$$

$$\Delta_{01}^{(2)} = Z^2\left(2\frac{\mid\langle\Psi_0^0|\, v\,|\Psi_1^0\rangle\mid^2}{E_1^0 - E_0^0} + \sum_{k>1}\left(\frac{\mid\langle\Psi_k^0|\, v\,|\Psi_1^0\rangle\mid^2}{E_1^0 - E_k^0} - \frac{\mid\langle\Psi_k^0|\, v\,|\Psi_0^0\rangle\mid^2}{E_0^0 - E_k^0}\right)\right). \tag{S9}$$

This demonstrates the parabolic evolution of the Stark shift as a function of the charge located in the center of the molecule.

The first order correction $\Delta_{01}^{(1)}$ can be written as

$$\Delta_{01}^{(1)} = \int \mathrm{d}\boldsymbol{r}\Psi_1^{0*}(\boldsymbol{r})V(\boldsymbol{r})\Psi_1^0(\boldsymbol{r}) - \int \mathrm{d}\boldsymbol{r}\Psi_0^{0*}(\boldsymbol{r})V(\boldsymbol{r})\Psi_0^0(\boldsymbol{r}) = Z\int \mathrm{d}\boldsymbol{r}\frac{\Delta\rho_{01}(\boldsymbol{r})}{\mid \boldsymbol{r} \mid}, \tag{S10}$$

where $\Delta\rho_{01}(\boldsymbol{r})$ is the difference of the total electron densities between the states $|\Psi_0^0\rangle$ and $|\Psi_0^1\rangle$

$$\Delta\rho_{01}(\boldsymbol{r}) = N\int \mathrm{d}\boldsymbol{r}_2\mathrm{d}\boldsymbol{r}_3\cdots\mathrm{d}\boldsymbol{r}_N\left(\left|\Psi_1^0\left(\boldsymbol{r},\boldsymbol{r}_2,\boldsymbol{r}_3\cdots\boldsymbol{r}_N\right)\right|^2 - \left|\Psi_0^0\left(\boldsymbol{r},\boldsymbol{r}_2,\boldsymbol{r}_3\cdots\boldsymbol{r}_N\right)\right|^2\right), \tag{S11}$$

where N is the total number of electron in the molecules.

The quadratic coefficient cannot be estimated in a simple way, since it requires the knowledge of the higher lying excited states which is out of reach of standard TD-DFT calculations.

Supplementary References

[1] Amati, M., Stoia, S. & Baerends, E. The electron affinity as the highest occupied anion orbital energy with a sufficiently accurate approximation of the exact kohn-sham potential. *J. Chem. Theory Comput.* **16**, 443–452 (2020).

[2] Swart, I., Sonnleitner, T. & Repp, J. Charge state control of molecules reveals modification of the tunneling barrier with intramolecular contrast. *Nano Lett.* **11**, 1580–1584 (2011).

[3] Davydov, A. *Quantum Mechanics, 2nd ed.* (Pergamon Press, Oxford, 1976).
